# Hallucinations as the Primary Presenting Feature in "Folie à Famille": A Case Report

**DOI:** 10.7759/cureus.92209

**Published:** 2025-09-13

**Authors:** Monalisa Boro, Vivek Saharan, Pranjal Dey

**Affiliations:** 1 Psychiatry, All India Institute of Medical Sciences, Guwahati, IND

**Keywords:** delusion of parasitosis, folie a deux, folie a famille, hallucinations, shared psychotic disorder

## Abstract

Shared psychotic disorder (SPD) is a rare phenomenon. This case report describes a 60-year-old man (primary subject) who first developed visual hallucinations, which he claimed to see *small, white insects*, thread-like, about half a centimeter, emerging from the folds of his wife’s skin near the elbow crease, wriggling, and then vanishing. Following his persistent suggestions, his wife (secondary subject) and later their son also developed similar hallucinations. Subsequently, delusion of parasitosis developed secondary to hallucinations in all of them. The family lives in a sparsely populated locality in close association with each other, and mostly stay indoors. The family holds strong traditional health beliefs. On detailed evaluation, no history of prior mental disorders was noted. Physical examination revealed no signs of infestation at the reported sites, and investigations did not indicate any underlying medical condition. The symptoms subsided significantly after treating them with risperidone (up to 6 mg) and trihexyphenidyl (2 mg) over a period of four weeks. This case highlights the occurrence of hallucinations as the primary symptom in folie à famille, followed by the development of delusions of parasitosis. The existing literature on folie à famille is limited, and this case report will encourage further research to clarify the exact phenomenology of this rare presentation.

## Introduction

Psychopathology of shared psychotic disorder (SPD) is not well understood, as it is a rare phenomenon. Jules Baillarger first reported this condition in 1860 [1]. The term *folie à deux* was later described by Lasegue and Falret in 1877 [2]. Gralnick defined it as “the transfer of delusional beliefs and/or abnormal behaviour from one person (primary) to another (secondary) or one person to several others, who have been in close association with the primary affected person” [3]. Hence, it is also referred to as association psychosis [3]. Folie à famille is a form of folie à deux where more than two members of the family share the same delusions [4]. The delusions are transferred from the dominant partner, who first develops them, to the more passive and submissive partner [4]. However, the exact cause of SPD is still unknown, but a long duration of relationship, social isolation, introverted and emotionally immature personality traits, and female gender are some of the risk factors [1]. Folie à deux cases account for 1.7%-2.6% of psychiatric hospital admissions [1]. The prevalence of folie à deux is difficult to determine due to its rare occurrence. This condition is often underdiagnosed and missed in clinical practice.

We are reporting a case of folie à famille in a family of three socially isolated members. Visual hallucination in the husband was the first presenting symptom, followed by similar symptoms in his wife and son. This case illustrates how delusion of parasitosis developed secondary to hallucinations in all family members. Hallucinations as the primary presenting symptom in SPD (folie à famille) are rarely reported, which prompted us to report this case.

## Case presentation

The patient was a 60-year-old man engaged in farming, and the patient's wife was 50 years old and a homemaker. The patient and the patient's wife had studied up to the primary level. They had been married for over three decades and lived with their 22-year-old son in an isolated village in northeastern India. Their household income was modest and irregular, mostly dependent on seasonal crop yield. Their surroundings were sparsely populated, with neighbors living half a mile apart. The family occasionally participated in social gatherings but mostly stayed indoors. Mobile phone usage was limited, and television served as their main source of information and entertainment. The family held traditional health beliefs and sought medical attention only when absolutely necessary.

The patient demonstrated an anankastic-anxious premorbid personality, characterized by hypervigilance, health-related preoccupations, and a strong sense of responsibility toward his family. Since his brother’s sudden death two years ago, he had shown increased ruminative thinking and hypochondriacal tendencies, often misinterpreting benign bodily sensations as signs of serious health conditions. The patient's wife demonstrated a submissive-dependent premorbid personality, while their son exhibited anxious-avoidant personality traits.

The patient's wife began experiencing intermittent, dull aching pain in her left elbow about a year before the current presentation, which gradually worsened due to repeated manual labor such as lifting heavy objects. She never sought formal medical advice for this condition, dismissing it as age-related. Approximately three months before the current clinical presentation, the couple were seated in their veranda after completing their daily chores, when the patient's wife began massaging her sore elbow with mustard oil. The patient reported noticing what he described as *small, white, thread-like insects*, about half a centimeter in length, emerging from the folds of her skin near the elbow crease, wriggling, and then vanishing. Alarmed, the patient immediately wiped the site and insisted that his wife had a parasitic infection. The patient's wife was puzzled and reported feeling nothing unusual. He became intensely concerned and grew increasingly restless. Over the following days, the patient repeatedly examined her elbow, collecting flakes of skin and dust as *specimens* and insisting that something was *burrowing inside her*.

Within a week, the patient's wife reported hesitantly that she too saw fleeting white specks coming out of her skin. She gradually began feeling sensations of crawling and occasional pin-pricking on the same elbow. These sensations became more vivid in the evenings. Two weeks later, their son also began reporting similar experiences, avoided touching his mother’s elbow, and sat apart during meals. He remarked, “They are tiny. Maybe they live under her skin. I saw them too.” However, neither the father nor the son developed tactile hallucinations.

By the fourth week, all three family members engaged in ritual cleaning, applying kerosene and salt to *disinfect *the elbow, scrubbing bed linens daily, and avoiding any social contact. The patient stopped visitors from entering their home, burned three sets of bedclothes, and destroyed old clothes he believed were *infested*. Despite reassurances from their village health worker and a general physician who found no visible signs of infestation, the family remained convinced. All three family members presented to the Dermatology outpatient services of a tertiary care hospital, from where they were referred to the Psychiatry outpatient department (OPD). On detailed evaluation, there was no history of prior mental illness, substance abuse, or trauma in any of the family members or their extended relatives. There was no history of major medical illness or parasitic infection in any of them. Mental status examination revealed visual hallucination, delusion of parasitosis, intact cognitive functions, and poor insight (grade 1) in all three individuals. Tactile hallucinations were present only in the patient's wife. The 18-item Brief Psychiatric Rating Scale (BPRS) scores were 58, 62, and 54 (out of 126) for the patient, the patient's wife, and their son, respectively. Physical examination revealed no signs of infestation at the reported sites, and vital signs, including blood pressure, respiratory rate, pulse rate, oxygen saturation (SpO₂), and body temperature, were within normal limits. Laboratory investigations were within normal limits and did not reveal any underlying medical conditions. All three were started on risperidone 2 mg along with trihexyphenidyl 2 mg once daily. Risperidone was gradually titrated to 4 mg once daily after six days, and further increased to 6 mg once daily at the two-week follow-up. All three were prescribed the same dosage as they presented together with similar psychopathology and continued on the same regimen at the four-week follow-up. The primary case was advised to maintain distance from the secondary cases until each follow-up. The family was also psychoeducated regarding their condition. Hallucinations resolved first in the patient, followed by his wife and their son, with a significant reduction in BPRS scores (15, 18, and 10 for the patient, the patient's wife, and their son, respectively) at the four-week follow-up. Delusion was the last symptom to resolve in all three. However, they did not return for follow-up after that.

## Discussion

In the present case, the inducer (the husband) appeared to have transferred his hallucination to the recipients (his wife and son) through persistent insistence. Looking back at the original theories formulated by Lasegue and Falret, it has been noted that women are more suggestible, due to their submissive and dependent roles in traditional families [2,5]. The family’s close association, nuclear structure, and geographic isolation likely contributed to the phenomenon [6]. In this case, hallucinations were the first presenting symptoms in all three subjects, while the delusion of parasitosis developed secondarily to the hallucinations. A systematic review revealed that almost all (100%) of the patients presented with delusional beliefs, but it was not clear whether the delusions were primary or developed secondary to hallucinations [7]. In the primary cases, persecutory and grandiose delusions were commonly encountered [6]. Hallucinations could develop in secondary cases but might not be shared by the primary [6]. Auditory hallucinations were reported in about 50% of primary patients, compared to thirty percent of secondary patients, in whom symptoms tended to be milder and shorter in duration [6,8]. After auditory hallucinations, somatic and visual hallucinations were commonly observed [6]. In this case, the primary patient had visual hallucinations, while the secondary (the wife) had both visual and tactile hallucinations. Hallucinations in SPD are often shared through suggestion from the inducer to the recipient, as demonstrated in this case [9]. Emotional closeness of the individuals and social isolation play a crucial role in developing hallucinations [10].

SPD was classified under *Induced Delusional Disorder* (F24) in ICD-10, but it did not mention hallucinations as primary presenting symptoms or as a major criterion for the diagnosis [11]. As per ICD-11, patients presenting with symptoms that do not fulfill the criteria for any specific psychotic disorder are classified under *other primary psychotic disorders* [12]. Similarly, SPD is not recognized as a separate diagnosis in the *Diagnostic and Statistical Manual of Mental Disorders, Fifth Edition, Text Revision* (DSM-5-TR); it falls under *other specified schizophrenia spectrum and other psychotic disorders*, which emphasizes the transfer of delusional beliefs, but not hallucinations, from a primary case to recipients [13]. Shared psychotic disorder does not necessarily involve only delusions; it can also include hallucinations [6]. Hence, the essential criteria required for the diagnosis within current classification systems are insufficient. To avoid missing this diagnosis, clinicians should be encouraged to routinely screen for psychotic symptoms in the family members of patients with psychotic disorders.

## Conclusions

Although hallucinations frequently occur in SPD, they are not currently included in the diagnostic criteria (ICD-11 and DSM-5-TR), and therefore should be considered in future versions of diagnostic classification systems. Also, the classification systems do not specify the exact criteria for the diagnosis of SPD as a distinct or separate diagnosis. There is a lack of literature on *folie à famille*, and hence, further research is needed to clarify the phenomenology of this rare presentation.
